# ﻿Molecular and morphological evidence reveals hidden new taxa in *Ochlodesochraceus* (Bremer, 1861) (Lepidoptera, Hesperiidae, Hesperiinae) from China

**DOI:** 10.3897/zookeys.1169.102322

**Published:** 2023-07-14

**Authors:** Lijuan Zhu, Yongxiang Hou, Hideyuki Chiba, Yohei Osada, Zhenfu Huang, Sergey Yu. Sinev, Min Wang, Xiaoling Fan

**Affiliations:** 1 Department of Entomology, College of Plant Protection, South China Agricultural University, Guangzhou, 510642, China South China Agricultural University Guangzhou China; 2 B. P. Bishop Museum, Honolulu, Hawaii, 96817-0916, USA B. P. Bishop Museum Honolulu United States of America; 3 Osaka Museum of Natural History, 1-23 Nagai Park, Higashisumiyoshi-ku, Osaka, 546-0034, Japan Osaka Museum of Natural History Osaka Japan; 4 School of Life Science and Engineering, Southwest University of Science and Technology, Mianyang, 621010, China Southwest University of Science and Technology Mianyang China; 5 Zoological Institute of the Russian Academy of Sciences, Universitetskaya Emb. 1, 199034, Saint-Petersburg, Russia Zoological Institute of the Russian Academy of Sciences Saint-Petersburg Russia

**Keywords:** Genitalia, Hubei, new species, phylogeny, Shaanxi, Sichuan, taxonomy, Zhejiang

## Abstract

Two new species of *Ochlodes* Scudder, 1872, *Ochlodespseudochraceus* Zhu, Fan & Wang, **sp. nov.** and *Ochlodescryptochraceus* Zhu, Fan & Chiba, **sp. nov.**, are found in China and described, and *Ochlodesrikuchina* (Butler, 1878) **stat. rev.** is restored. A lectotype is designated for *Pamphilaochracea* Bremer, 1861, and a neotype is designated for *Pamphilarikuchina* Butler, 1878. Overall, the two new species are similar to *Ochlodesochraceus* (Bremer, 1861). They, however, can be distinguished from the latter and other species in the genus: *O.pseudochraceus* has long radial spots in spaces R_3-5_, and the lateral process of the phallus gradually widens at the distal half in male genitalia; *O.cryptochraceus* has the lateral process of the phallus enlarged only at the distal tip. Based on the phylogenetic analyses of the mitochondrial COI gene, members of currently defined *O.ochraceus* are grouped into four clades. The genetic distances between *O.pseudochraceus* and *O.ochraceus*, *O.cryptochraceus* and *O.ochraceus*, *O.rikuchina* and *O.ochraceus*, and *O.pseudochraceus* and *O.cryptochraceus* are 3.2%, 2.1%, 1.9%, and 2.7%, respectively. Based on the molecular and morphological evidence, *O.pseudochraceus*, *O.cryptochraceus*, and *O.rikuchina* are treated to be distinct species. The adult habitus and male and female genitalia of the new species are illustrated as well as those of *O.ochraceus* and *O.rikuchina*.

## ﻿Introduction

The genus *Ochlodes* Scudder, 1872, described with *Hesperianemorum* Boisduval, 1852 as the type species, belongs to the family Hesperiidae and is distributed in the Oriental, Palearctic, and Nearctic regions. Evans (1949, 1955) recognized 16 species and 23 subspecies within the genus. Chiba and Tsukiyama (1996) revised the genus based on wing pattern, male genitalia, and geographical distribution. A total of 23 species and 17 subspecies were recognized and classified into the following four groups: *venata* complex, *subhyalina* group, *bouddha* group, and a miscellaneous group. Among these, five species are distributed in the Nearctic region and the remainder in the Palearctic and Oriental regions.

China is the most species-rich area for the genus *Ochlodes*, with 16 species recorded to date (Burns 1992; Chiba and Tsukiyama 1996; Cao et al. 2019; Hsu et al. 2006; Hsu et al. 2019). During our revisional study of the genus, we observed that 16 specimens of *O.ochraceus* collected in China (Shaanxi, Sichuan, Zhejiang, and Hubei), Japan, and Russia (type locality) were grouped into four clades based on the COI gene, corresponding to two samples from Russia, six samples from Japan, three samples from Zhejiang, China, and five samples from Shaanxi, Sichuan, and Hubei, China. Based on careful examination, we found that the specimens of the four clades differed in genitalic morphology, and those from Zhejiang and other localities in China differed from all other species in the genus. Therefore, four distinct species can be recognized, and the specimens from Shaanxi, Sichuan, Hubei, and Zhejiang in China represent two new species.

## ﻿Materials and methods

### ﻿Morphological study

The specimens examined in this study were collected using an insect net and deposited at the South China Agricultural University (**SCAU**), Guangzhou, China. The specimens in the following institutional and private collections were also examined: Northeast Forestry University (**NEFU**), Harbin, China; private collection of Hideyuki Chiba (**HC**), Fukuoka, Japan; Osaka Museum of Natural History (**OMNH**), Osaka, Japan; Hokkaido University Museum (**HUM**), Hokkaido, Japan; Leibniz Institute for the Analysis of Biodiversity Change, Zoological Research Museum Alexander Koenig (Zoologisches Forschungsmuseum Alexander Koenig, **ZFMK**), Bonn, Germany; Zoological Institute of Russian Academy of Sciences (**ZIN**), Saint Petersburg, Russia; Natural History Museum, London, United Kingdom (**NHMUK**, formerly BMNH). Images of the type of *O.rikuchina* were used with permission (copyright: Trustees Natural History Museum, photograph R. Crowther). All adult photographs were captured using a SONY DSC-RX100 camera. The abdomens were removed and macerated in 10% NaOH solution to examine the male and female genitalia. Genitalia were photographed using the Keyence VHX-5000 ultra-depth of field 3D microsystem. The wing venation was examined according to the method Hou et al. (2021) outlined and photographed using a smartphone. All photographs were processed by Abode Photoshop CC and Abode Illustrator CC 2018. The terminology for adults and genitalia follows Chiba and Tsukiyama (1996) and Fan et al. (2010).

### ﻿Molecular analysis

Based on the classification of Chiba and Tsukiyama (1996), 29 specimens of *Ochlodes* were sampled as ingroups, representing two species placed in the miscellaneous group: *Ochlodeslinga* Evans, 1939 and *O.ochraceus*, and five species placed in all other species groups. Whenever possible, samples from the type localities or near the type localities were included for previously described taxa. The COI barcodes of all 29 specimens were sequenced, and the sequences were deposited in GenBank. The sequence information of two species of *Hesperia* (*H.meskei* and *H.attalus*) was downloaded from GenBank (https://www.ncbi.nlm.nih.gov) as outgroups based on prior information (Yuan et al. 2015b). Detailed information on materials and accession numbers is provided in Table 1. Our previous studies referred to details of the DNA extraction, amplification, and sequencing protocols (Huang et al. 2019a; Hou et al. 2021). Genetic distances were calculated using Kimura 2-parameter models in MEGA 7.0 (Kumar et al. 2016). Phylogenetic trees were constructed using maximum likelihood (ML) and Bayesian inference (BI) methods. ML analyses were performed using IQ-TREE 2.2.1 (Minh et al. 2020) on a local computer. The data were partitioned into codon positions and models (1st: HKY+F+G4, 2nd: TN+F+I, and 3rd: F81+F+I) were selected using ModelFinder (Kalyaanamoorthy et al. 2017) in IQ-TREE 2.2.1 (Minh et al. 2020). The nucleotide substitution models were estimated under the Bayesian Information Criterion (BIC) with FreeRate heterogeneity, which relaxes the assumption of gamma-distributed rates. Both Ultrafast bootstrap (UFBoot) (Minh et al. 2013) and SH-aLRT branch test (Guindon et al. 2010) were performed with 1000 replicates to evaluate branch support, and the tree with the highest likelihood was selected. The BI analyses were performed using MrBayes v. 3.2.6 on CIPRES Science Gateway 3.3 (http://www.phylo.org/) (Miller et al. 2010) with Markov Chain Monte Carlo (MCMC) randomization in MrBayes using XSEDE 3.2.6 (Ronquist et al. 2012). Two independent runs were performed, and the starting tree was set to a random tree. Four Markov chains (three hot chains and one cold chain) ran 5 × 10^6^ generations simultaneously, sampling every 1000 generations, with the first 25% of sampled trees discarded as burn-in. Tracer v. 1.7.2 (Rambaut et al. 2018) was used to determine the standard deviation of the split frequency value, which was < 0.01, and the effective sample size (ESS) > 200, indicating that the runs reached stationarity. Bayesian posterior probabilities (PP) were used to evaluate branch support. Trees were visualized using FigTree v. 1.4.4 (http://tree.bio.ed.ac.uk/software/figtree/).

**Table 1.** Voucher information and GenBank accession numbers for specimens used in this study.

Taxon	Data	Locality	Voucher number	Accession number
*Ochlodesvenatus*	VIII. 2009	Beijing, China	SCAU_He2718	OQ452925
*Ochlodesvenatus*	VII.2018	Heilongjiang, China	SCAU_He2602	OQ452926
*Ochlodessimilis*	VI. 2021	Songpan, Sichuan, China	SCAU_He2650	OQ452930
*Ochlodessimilis*	VI. 2010	Beijing, China	SCAU_He2658	OQ452929
*Ochlodessagittus*	VII.2018	Kangding, Sichuan, China	SCAU_He2613	OQ452932
*Ochlodessagittus*	VI. 2021	Songpan, Sichuan, China	SCAU_He2647	OQ452931
*Ochlodesbouddha*	VI. 2019	Yaan, Sichuan, China	SCAU_He2620	OQ452947
*Ochlodesbouddha*	VII.2019	Ankang, Shaanxi, China	SCAU_He2685	OQ452946
*Ochlodessubhyalinus*	VIII. 2018	Hanzhong, Shaanxi, China	SCAU_He2646	OQ452927
*Ochlodessubhyalinus*	VI. 2017	Tianshui, Gansu, China	SCAU_He2617	OQ452928
*Ochlodeslinga*	V. 2018	Hanzhong, Shaanxi, China	SCAU_He2696	OQ452941
*Ochlodeslinga*	VI. 2018	Baoji, Shaanxi, China	SCAU_He2697	OQ452940
*Ochlodeslinga*	V. 2018	Baoji, Shaanxi, China	SCAU_He2698	OQ452939
*Ochlodesochraceus*	VI. 2019	Shennongjia, Hubei, China	SCAU_He2605	OQ452945
*Ochlodesochraceus*	VI. 2018	Yulin, Shaanxi, China	SCAU_He2632	OQ452944
*Ochlodesochraceus*	VI. 2019	Shennongjia, Hubei, China	SCAU_He2677	OQ452943
*Ochlodesochraceus*	VI. 2021	Baoji, Shaanxi, China	SCAU_He2678	OQ452942
*Ochlodesochraceus*	VII. 2022	Mianyang, Sichuan, China	SCAU_He2746	OQ749886
*Ochlodesochraceus*	VIII. 2016	Ningbo, Zhejiang, China	SCAU_He2614	OQ452935
*Ochlodesochraceus*	VIII. 2016	Ningbo, Zhejiang, China	SCAU_He2637	OQ452934
*Ochlodesochraceus*	VIII. 2016	Ningbo, Zhejiang, China	SCAU_He2676	OQ452924
*Ochlodesochraceus*	VII.2022	Ueda-shi, Japan	SCAU_He2729	OQ452937
*Ochlodesochraceus*	VII.2021	Ueda-shi, Japan	SCAU_He2730	OQ452933
*Ochlodesochraceus*	VII. 2017	Japan	SCAU_He2734	OQ749884
*Ochlodesochraceus*	VII. 2017	Japan	SCAU_He2736	OQ749885
*Ochlodesochraceus*	VII. 2021	Iwate-ken, Japan	SCAU_He2726	OR058650
*Ochlodesochraceus*	V. 1990	Aichi-ken, Japan	SCAU_He2727	OR058651
*Ochlodesochraceus*	III.2018	Primorsky Kray, Russia	SCAU_He2728	OQ452938
*Ochlodesochraceus*	VII.2015	Primorsky Kray, Russia	SCAU_He2732	OQ452936
*Hesperiameskei*	X.1997	Florida, United States	CSU-CPG-LEP001666	GU685651
*Hesperiaattalus*	III.2002	California, United States	AAE3790	GU685041

Combining DNA sequence data with other kinds of characters produces a more precise taxonomic framework (DeSalle et al. 2005). DNA barcoding helps recognize cryptic species (Nolasco and Valdez-Mondragón 2022). In this study, three different criteria were adopted, namely, morphological characters, monophyly, and genetic distance, for species delimitation based on the unified species concept described by de Queiroz (2005). If two taxa are recovered as monophyletic and have consistent morphological differences, and the genetic distance between them is not less than 0.8% (the genetic distance between *Ochlodessimilis* and *O.sagittus*, which are morphologically two clearly distinct species with sympatric distribution), they are treated as two distinct species.

## ﻿Results and discussion

### ﻿Phylogenetic analysis

**Figure 1.**
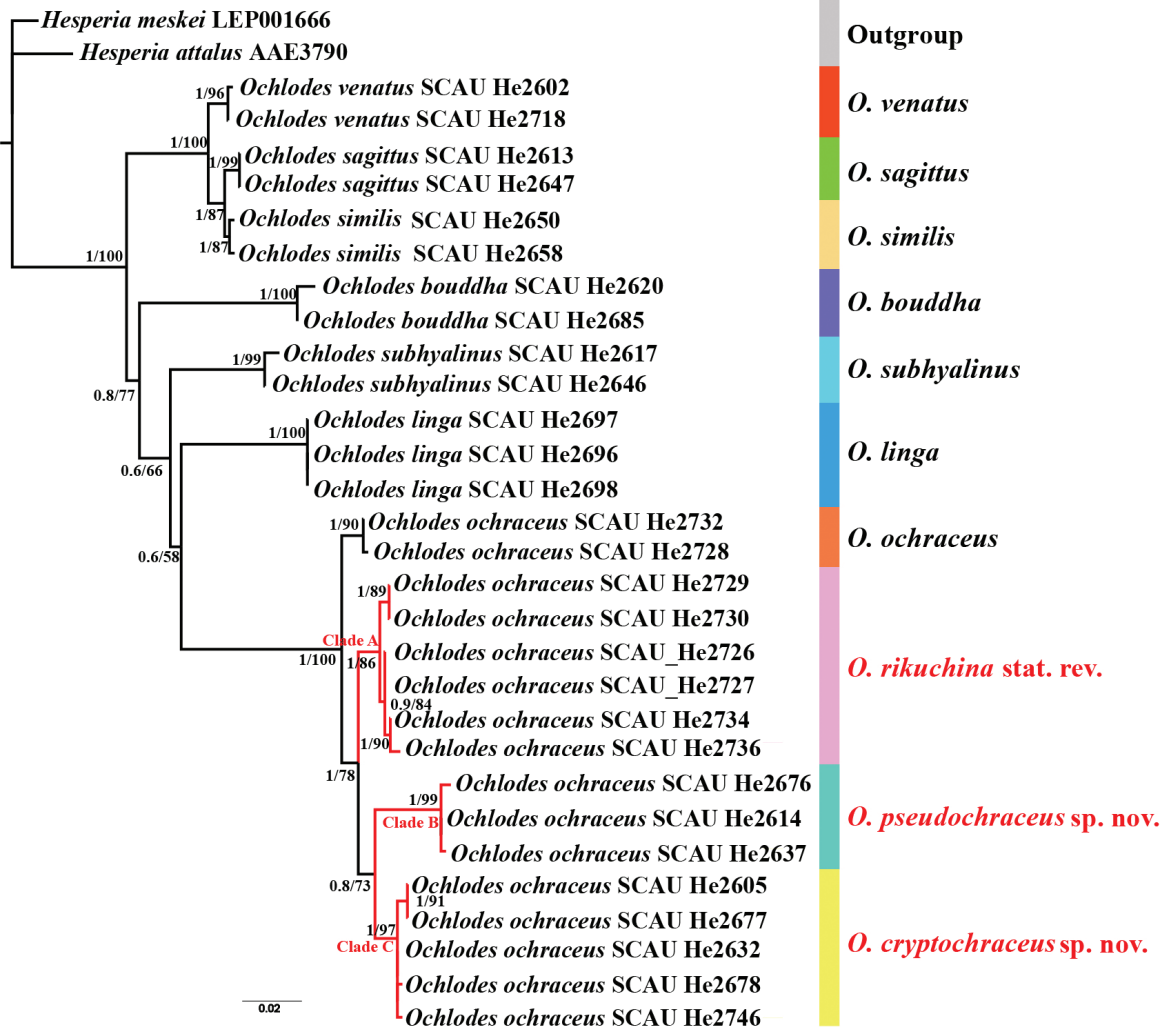
Phylogenetic tree of *Ochlodes* based on COI barcode region, using the ML and BI methods. Values at nodes represent the posterior probabilities (PP) of BI analyses and Ultrafast bootstrap values (UFBoot) of the ML analyses.

The phylogenetic tree (Fig. 1) constructed using the COI barcoding region shows that the members of the genus *Ochlodes* are clustered together with strong support (PP/UFBoot = 1/100). The following five lineages are recognized: 1) *venatus* complex, including *O.venatus*, *O.similis*, and *O.sagittus*; 2) *O.bouddha*; 3) *O.linga*; 4) *O.subhyalinus*; and 5) *O.ochraceus*. The miscellaneous group of Chiba and Tsukiyama (1996) is divided into two distant clades. The 16 samples of *O.ochraceus* are clustered together and recovered in four subclades: *O.ochraceus* from Primorsky Kray, Russia; clade A from Japan; clade B from Zhejiang, China; and clade C from Shaanxi, Sichuan, and Hubei in China. The genetic distance range among species calculated with COI barcode is 0.8–9.9%, of which the distance range among the three species within *venatus* complex is 0.8–1.6%, the distance among the four subclades of *O.ochraceus* is 1.9% between *O.ochraceus* and clade A, 3.2% between clade A and clade B, 2.1% between clade A and clade C, 3.2% between *O.ochraceus* and clade B, 2.1% between *O.ochraceus* and clade C, and 2.7% between clade B and clade C (Table 2). The traditional *O.ochraceus* clade, characterized by a wider and darker wing margin, is named the *ochraceus* complex.

**Table 2.** Genetic distances among *Ochlodes* species based on COI barcodes.

	1	2	3	4	5	6	7	8	9
*O.venatus*									
*O.similis*	0.015								
*O.sagittus*	0.016	0.008							
*O.bouddha*	0.068	0.070	0.072						
*O.linga*	0.066	0.064	0.067	0.065					
*O.subhyalinus*	0.059	0.058	0.061	0.071	0.059				
*O.ochraceus*	0.069	0.070	0.073	0.086	0.073	0.067			
*O.rikuchina*	0.080	0.082	0.085	0.099	0.082	0.076	**0.019**		
*O.pseudochraceus*	0.076	0.078	0.081	0.084	0.079	0.070	**0.032**	**0.032**	
*O.cryptochraceus*	0.075	0.076	0.080	0.086	0.076	0.071	**0.021**	**0.021**	**0.027**

### ﻿Taxonomy of the *ochraceus* complex and relatives

**Figure 2.**
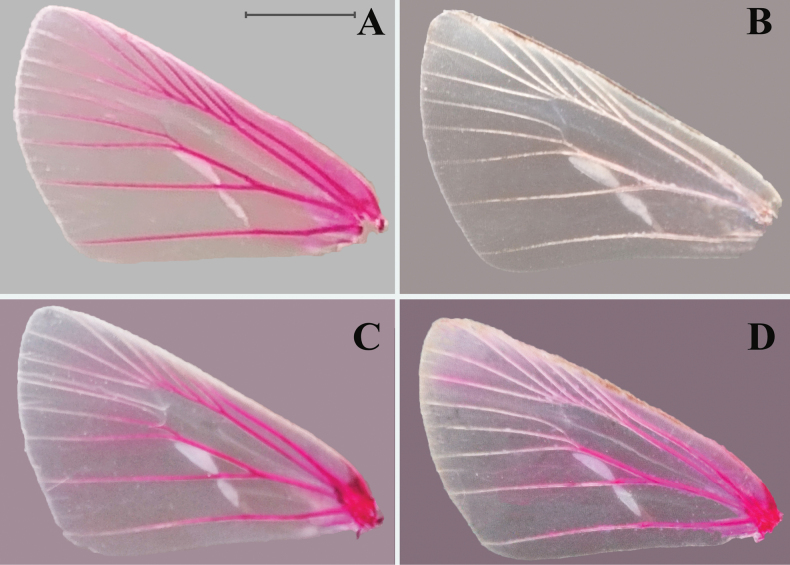
Wing venation and stigma of *ochraceus* complex. **A***O.pseudochraceus* sp. nov., holotype, Zhejiang, SCAU He2614 **B***O.cryptochraceus* sp. nov., holotype, Hubei, SCAU He2605 **C***O.rikuchina* stat. rev., neotype, Iwate, SCAU He2726 **D***O.ochraceus*, Russia, SCAU He2728. Scale bar: 0.5 cm.

**Figure 3.**
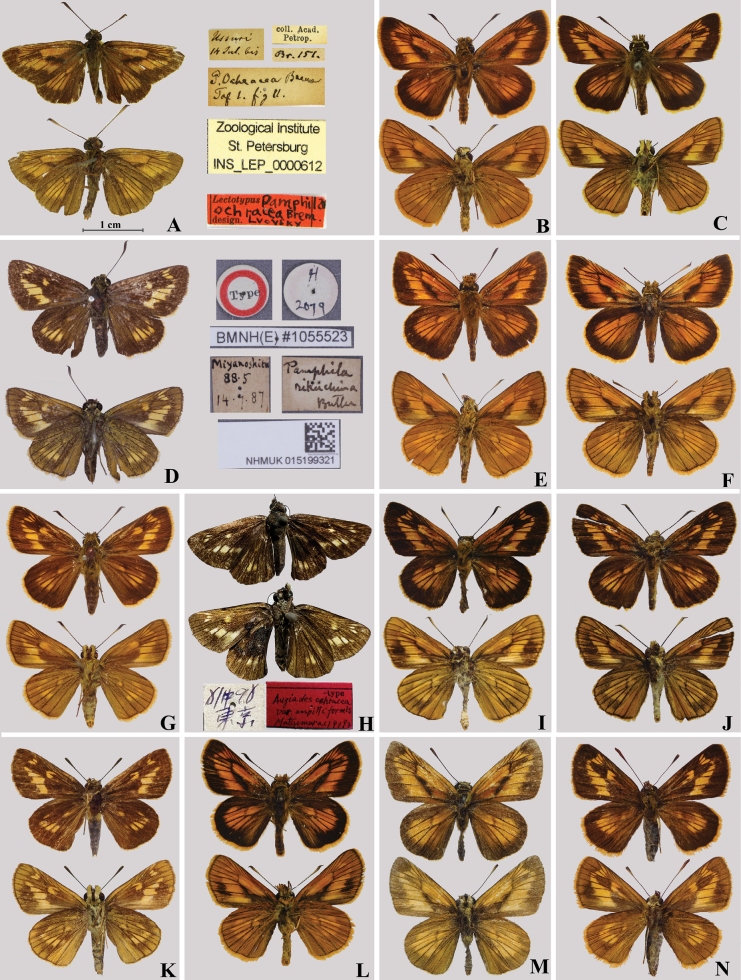
Adults of four *Ochlodes* species **A–C***O.ochracea***A** lectotype, male, Primorsky Kray, Russia **B** male, Primorsky Kray, Russia, SCAU He2728 **C** male, Amur, Russia **D–G***O.rikuchina* stat. rev. **D** female, Miyanoshita, Japan (NHMUK) **E** neotype, male, Iwate, Japan (designated herein), SCAU He2726 **F** male, Japan, SCAU He2736 **G** female, Japan, SCAU He2727 **H**Augiadesochraceavar.ampittiformis. holotype, female, Tokyo, Japan **I–K***O.pseudochraceus* sp. nov. **I** holotype, male, Zhejiang, SCAU He2614 **J** paratype, male, Zhejiang, SCAU He2676 **K** paratypes, female, Zhejiang, SCAU He2637 **L–N***O.cryptochraceus* sp. nov. **L** holotype, male, Hubei, SCAU He2605 **M** paratype, male, Shaanxi, SCAU He2678 **N** paratypes, female, Shaanxi, SCAU He2632.

We examined the syntype of *O.ochraceus* from Primorsky Kray, Russia (https://www.zin.ru/collections/Lepidoptera), deposited in ZIN (Fig. 3A). Despite the label indicating its status as a lectotype, such a designation has not been published. Therefore, we herein designate the male specimen as the lectotype. Apart from the lectotype, 12 other specimens of *O.ochraceus* (five from Primorsky Kray, two from Amur, Russia, and five from Heilongjiang, China) were examined. We also examined the specimen whose labels indicate “Type, the locality Miyanoshita” at NHMUK (Fig. 3D) and 55 specimens of *O.rikuchina* from Japan. All the taxa in the *ochraceus* complex share consistent and distinct morphological characters (Table 3). *Ochlodesochraceus* from Russia and *O.rikuchina* (clade A) from Japan share the following characters in male genitalia: the dorsodistal process is finger-like, the ventrodistal process of the valva is broad and round distally, and the lateral process of the phallus is not enlarged. In *O.ochraceus*, however, the tegumen extends distally, and the uncus is wide. In contrast, in *O.rikuchina*, the tegumen does not extend distally, and the uncus is narrow. Members of clade B can be distinguished from the other taxa by their wing patterns and male genitalia. The spots in spaces R_3_–R_5_ on the forewing upper side are short and radial and away from the discocellular vein, and the lateral long process of the phallus is gradually widened with a row of small spines along the dorsal margin. In the other taxa of the complex, these spots are long and reach the discocellular vein, and the process of the phallus is only enlarged at the distal tip (clade C) or not significantly widened (clade A). In addition, the stigma of these taxa is divided into three parts: the first part in space CuA_1_ and the second in space CuA_2_ are markedly different, whereas the third part in space CuA_2_ is vague. The first and the second parts of *O.ochraceus* are crescent- and spindle-shaped, respectively, differing from those in the other taxa (Fig. 2). Therefore, we believe that the currently recognized *O.ochraceus* is not a single species but includes hidden species. Based on morphological characters and molecular evidence, we describe clades B and C as two new species below: *O.pseudochraceus* Zhu, Fan & Wang, sp. nov. and *O.cryptochraceus* Zhu, Fan & Chiba, sp. nov. Additionally, clade A is recognized as a valid species, and *O.rikuchina* stat. rev. is restored.

**Table 3.** Comparison of morphological differences among four species of *ochraceus* complex in *Ochlodes*.

	*pseudochraceus*	*O.cryptochraceus*	*rikuchina*	*O.ochraceus*
**Color of wing margin**	dark	dark	pale brown	pale brown
**Stigma**	thin and long, not aligned at CuA_2_ vein.	thick and long, not aligned at CuA_2_ vein.	thick and short, aligned at CuA_2_ vein.	thick and short, aligned at CuA_2_ vein.
**Saccus**	thin and long	thin and long	thin and short	thick and short
**Phallus**	lateral process gradually enlarged at distal half and serrated, not reaching the tip of phallus	lateral process only enlarged at the distal tip	lateral process not enlarged, distal half with serrated reaching the tip of phallus	lateral process not enlarged, distal half with serrated reaching the tip of phallus
**Uncus**	narrow	slightly narrow	narrow	wide
**Tegumen**	not extend distally	slightly extent distally	not extent distally	extend distally

In the previous studies, *O.ochraceus* has been recorded in Zhejiang, China (Tong 1993; Chou 1994; Yang et al. 1994; Chu et al. 2017). However, the specimens illustrated by Tong (1993), Chou (1994) (female), and Yang et al. (1994) are *O.linga*. We observed no specimens or photographs of true *O.ochraceus* collected in Zhejiang. Similarly, the specimens illustrated in most previous studies (Chiba and Tsukiyama 1996: pl. 1 figs 20, 22; Cai et al. 2011; Yuan et al. 2015a; Wu and Hsu 2017) are *Ochlodescryptochraceus* Zhu, Fan & Chiba, sp. nov., whereas the specimen illustrated in Chu et al. (2017) is *Ochlodespseudochraceus* Zhu, Fan & Wang, sp. nov.

Butler (1878) described *Pamphilarikuchina* based on an unstated number of specimens from Rikuchu, an old name of northeastern Japan which includes most of current Iwate and a part of Akita prefecture, erroneously naming it after “Rikuchin” from the handwriting of M. A. Fenton (Matsuda 1995). In addition, Butler (1878) did not illustrate this species, nor specify the sex of the specimen(s) he examined. However, his description, “primaries with two ochreous spots at the end of the cell (the upper one punctiform), secondaries with an arched series of five ochreous spots on the discal” is clear enough to recognize the type is of female. Evans (1949) mentioned a female type specimen from Japan. We examined the female specimen of *Pamphilarikuchina* deposited in NHMUK (Fig. 3D), which indicates that the female specimen collected from Miyanoshita in [18]87 is not the syntype examined by Butler (1878). Blanca Huertas (pers. comm.) conducted a thorough search at NHMUK, including the Evans’ reference collection, but she did not find any other specimen labelled as the type of this taxa or with Butler’s label, implying that the syntype(s) is likely lost. Considering this, a neotype designated for this name *rikuchina* is necessary to stabilize the taxon.

According to Article 75.3 of ICZN (1999), the exceptional need for this neotype designation, apart from the loss of the name-bearing syntype specimen(s), was based on the following: (1) The status of *O.rikuchina* (Butler, 1878) has not been settled, and it was treated as a synonym of *O.ochraceus* (Evans 1949; Chiba and Tsukiyama 1996; Yuan et al. 2015a) or as a subspecies of *O.ochraceus* (Kudrna 1974; Kawazoé and Wakabayashi 1976; Lee 1982). Our morphological and molecular studies show that *O.rikuchina* is a valid species. (2) This species can be distinguished from the other taxa in the *ochraceus* complex by the club of antenna being thin and long, the male genitalia having the tegumen that does not extend distally, the uncus being narrow, and the phallus with distal half of lateral process not enlarged. (3) The neotype should be a female specimen based on the origin description (Butler 1878), but it is difficult to identify species based on a female in Hesperiidae, given that most specimens in the genus *Ochlodes* are males. To secure the nomenclatural stability, we designated a male specimen from Iwate (type locality) as a neotype for *O.rikuchina* based on our morphological and molecular studies.

**Neotype designation**: Omorisawa, Isawa, Oshu-shi, Iwate prefecture, Japan, 31.VII.2010, S. Sakuratani leg// SCAU_He 2726// (SCAU) (Fig. 3E). For detailed description, see Taxonomy below.

Matsumura (1919) described Augiadesochraceavar.ampittiformis based on a single female specimen from Nakano, near Tokyo, Japan, which is currently deposited in HUM. According to Article 73.1.2 of ICZN (1999), we consider that this female specimen to be the holotype fixed by monotypy based on the statement of only ‘one female specimen’ provided in the original description. We examined the holotype of Augiadesochraceavar.ampittiformis (Fig. 3H) and considered that the characters of the original description, “both wings with much smaller spots, an indistinct tiny anterior spot on the discocellular, and two tiny spots respectively in the 4^th^ and 5^th^ interspaces”, represent only an individual variation of *O.rikuchina*. This was due to the size of the wing pattern of *O.rikuchina* being slightly variable among individuals. The upper spot in the discal cell is indistinct (Fig. 3H; Chiba and Tsukiyama 1996: pl 3 fig. 1) or ranges from a small dot (Kawazoé and Wakabayashi 1976) to a spot slightly smaller than the lower spot in the female (Fig. 3G); In contrast, in the other species of the *ochraceus* complex, the upper spot is not smaller than the lower spot. Therefore, we treat *ampittiformis* as a junior subjective synonym of *rikuchina*.

Lee (1982) noted that it seemed reasonable to regard the Korean population as the nominate subspecies ochraceus. After carefully examining the photographs, it is tentatively concluded that those illustrated in Lee (1982: pl. 63 fig. 243 excluding C, D) are of *O.ochraceus*. Further investigation, however, is required.

Chiba and Tsukiyama (1996) treated three subspecies of *O.venatus* sensu Evans (1949), *similis*, *sagittus*, and *hyrcana* (now *sylvanus*), as distinct species and placed them in the *venata* complex. We follow the treatment based on morphological characters and their sympatric distribution.

Morphological characters are considered inadequate for the identification of skipper butterflies (Ackery et al. 1999), and it is common, particularly in Hesperiidae, for a complex of sibling species with similar morphological characters to be recognized as one species (Hebert et al. 2004; Burns and Janzen 2005; Huang et al. 2019b; Huang 2021). As taxonomic and biological research progresses, the relationships among species become clearer, often resulting in the recognition of hidden taxa. Further study is required to investigate the possible sympatry of the new species with *O.ochraceus* in Zhejiang.

### ﻿Taxonomic accounts

#### ﻿ Ochlodes ochraceus

Taxon classificationAnimaliaLepidopteraHesperiidae

(Bremer, 1861)

5E32264B-FDCB-5EB7-98F6-0AD19AB19C6E

Figs 3A–C , 4G, H

Pamphila ochracea Bremer, 1861: 473 (type locality, original label: Ussuri). Lectotype (location: Primorsky Kray, Russia), designated herein.
Ochlodes ochracea : Evans 1949: 353; Chiba and Tsukiyama 1996: 16, pl 1 fig. 19; Tuzov et al. 1997: 129; Wang 1999: 286; Zhou and Zhu 2003: 196.
Ochlodes ochracea rikuchina : Lee 1982, pl 63 figs 243A, B.

##### Diagnosis.

Antenna with black and white stripes, club thick. Male genitalia (*n* = 5): tegumen extend distally; uncus wide; valva with dorsodistal process finger-like and round at tip, ventrodistal process widen and round; phallus with lateral process longer than subzonal sheath, distal half not enlarged and serrated reaching tip of phallus.

**Figure 4.**
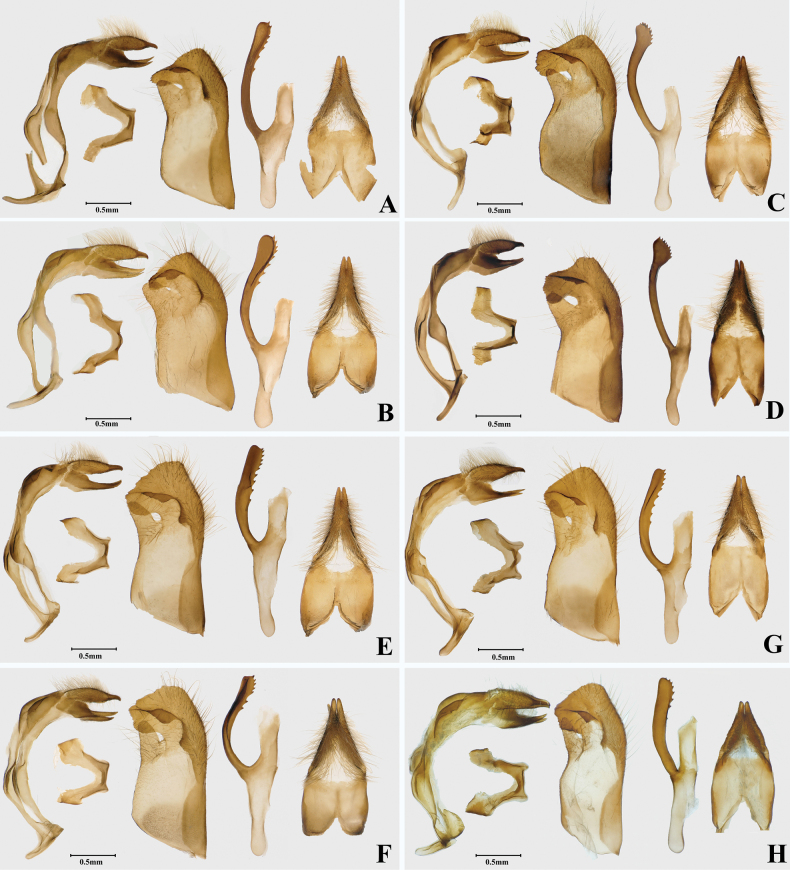
Male genitalia of four *Ochlodes* species **A, B***O.pseudochraceus* sp. nov. **A** holotype, male, SCAU He2614 **B** paratypes, male, SCAU He2676 **C, D***O.cryptochraceus* sp. nov. **C** holotype, male, SCAU He2605 **D** paratypes, male, SCAU He2678 **E**, **F***O.rikuchina* stat. rev. **E** male, SCAU He2726 **F** male, SCAU He2736 **G, H***O.ochraceus***G** male, SCAU He2728 **H** male, Amur, Russia.

##### Specimens examined.

***Lectotype***, ♂, Primorsky Kray, Russia (ZIN); 1♂, SCAU_He2728, 2.III.2018, Primorshy kray, Chuguevsky district, Russia (OMNH); 1♂, SCAU_He2731, 7.VII.2015, Primorshy kray, Spassky district, Russia (OMNH); 1♂, SCAU_He2732, 7.VII.2015, Primorsky Kray, Spassky district, Russia (OMNH); 1♂, 7.III.2018, Primorsky Kray, Chuguevsky district, Russia, Golovizin V. Col; 1♂, 7 VII 2016, Primorsky Kray, Chuguevsky district, Russia, Golovizin V. Col; 2♂, 6.VII.1974, Maoershan, Heilongjiang Province (NEFU); 1♂, Amur (ZFMK Lep153522); 1♂ VI 1927, Maoershan, Heilongjiang Province (ZFMK); 1♂, 7.VII.2016, Acheng district, Heilongjiang Province, (HC).

##### Distribution.

China (Heilongjiang, Jilin); Russia (Far East); Korea.

#### ﻿ Ochlodes rikuchina

Taxon classificationAnimaliaLepidopteraHesperiidae

(Butler, 1878) stat. rev.

F04C3D95-BFA0-57ED-9798-8DAFD2223712

**Figure 5.**
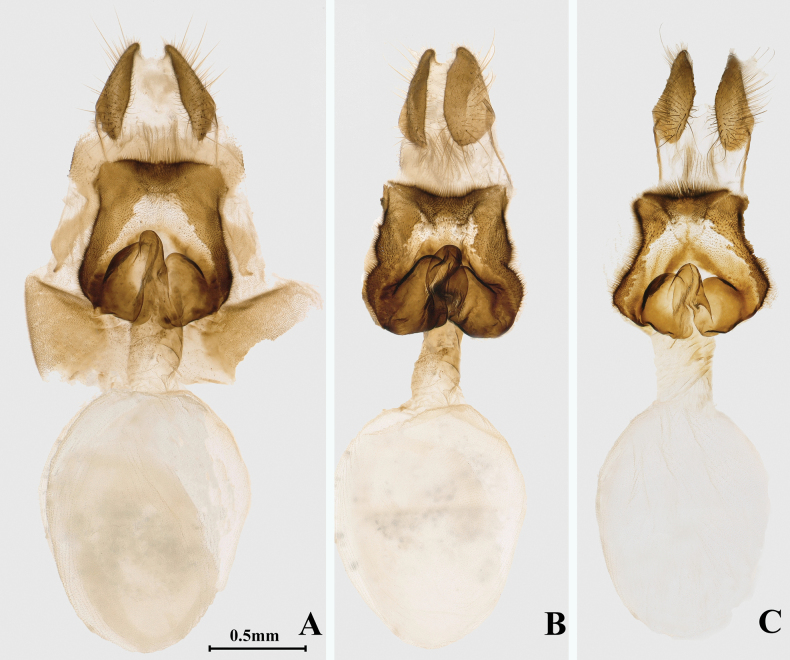
Female genitalia of four *Ochlodes* species **A***O.pseudochraceus* sp. nov., SCAU He2637 **B***O.cryptochraceus* sp. nov., SCAU He2678 **C***O.rikuchina* stat. rev., SCAU He2727.

Figs 3D–H , 4E, F , 5C

Pamphila rikuchina Butler, 1878: 285. Neotype ♂, designated herein (type locality: Oshu-shi, Iwate prefecture, Japan).
Augiades ochracea var. ampittiformis Matsumura, 1919: 737–738 (type locality: Nakano near Tokyo, Japan).
Ochlodes ochracea rikuchina : Kudrna 1974; Kawazoé and Wakabayashi 1976.

##### Specimens examined.

***Neotype***, ♂, SCAU_He 2726, 31.VII.2010, Omorisawa, Isawa, Oshu-shi, Iwate prefecture, Japan, S. Sakuratani leg; type, ♀, Miyanoshita, Japan (NHMUK//BMNH(E) #1055523); 1♂, SCAU_He 2729, 1♂, SCAU_He 2730, 12.VII.2021, Ueda-shi, Japan; 1♂, SCAU_He 2734, Japan; 1♀, SCAU_He2727, 17.VIII.1990, Aichi-pref., Mt. Naganoyama, Japan, Yamanaka leg.; 1♂, 31.VII.2010, S. Sakuratani leg., (HC); 1♂,7.VII.2001, Miyagi-pref., Ishinomaki, Japan, S. Sakuratani leg., (HC); 8♂, 7♀, 17.VII.1997, Gunma-pref., Mt. Haruna, Japan, H. Chiba leg., (HC); 1♂, 28.VII.1976, Gunma-pref., Hotaka, Japan, H. Chiba leg., (HC); 7♂, 1♀, 5.VI.1977, Tokyo, Itsukaichi, Japan, H. Chiba leg., (HC); 1♂, 30.V.1971, Tokyo, Okutama Japan, (HC); 1♂, 4.VII.1996, Tochigi-pref, Shinobara, Japan, S. Hashimoto leg., (HC); 1♂, 18.VII.1976, Nagano-pref, Lake Matsubara Japan, (HC); 2♂, 5.V.2007, Nagano-pref, Azumi Japan, (HC); 1♂, 7.VII.1996, Nagano-pref., Togakushi, Japan, A. Okubo leg., (HC); 1♂, 15.VII.1995, Nagano-pref., Kaida, Japan, Yamanaka leg., (HC); 3♂, 1♀, 2.VIII.1997, Nagano-pref., Kaida, Japan, Yamanaka leg., (HC); 4♂, 1♀, 17.VIII.1990, Aichi-pref., Mt. Naganoyama, Japan, Yamanaka leg., (HC); 1♂, 1♀, 20.VI.1995, Aichi-pref. Iwanami, Japan, Yamanaka leg., (HC); 1♂, 27.V.1990, Gifu-pref., Nagataki, Japan, H. Yamanaka leg., (HC); 1♂, 16.VI.1991, Okayama-pref., Niimi, Japan, Osaka leg., (HC); 2♂, 28.VI.1998, Okayama-pref., Kawakami Japan, (HC); 1♂, 20.V.1998, Miyazaki-pref., Takachiho, Japan, M. Murakami leg., (HC); 1♂, 12.VIII.1998, Miyazaki-pref., Takachiho, Japan, Murakami leg., (HC).

##### Diagnosis.

Antenna with black and white stripes, club thin and long. Male genitalia: tegumen not extend distally; uncus narrow; phallus with lateral process almost equal to subzonal sheath and not enlarged, distal half serrated.

##### Redescription.

Forewing length 15 ± 0.5 mm in males and 15.5 mm in females (Fig. 3E–G). Antenna longer than half length of forewing. Labial palpi: second segment porrect and covered with long brown hairs, third segment short.

**Male** (Fig. 3E, F). Forewing upper side: ground color brown with orange spots. Spots in spaces R_1_–R_5_ long radial and connected to discocellular vein; spots in space M_1_ to CuA_2_ form a broad brand, of which spot in space M_1_ very small; cell orange; stigma black-grey, thin and short. Hindwing upper side: ground color same as forewing, central part including cell and spaces Rs-CuA_2_ orange. Wing under side: ground color orange-yellow, with spots orange, and blurred.

**Female** (Fig. 3G). Different from male in reduced spots in spaces R_1_, _2_; only a couple of cell spots present on forewing; cell spot and spots in spaces Rs-CuA_2_ short and small on hindwing.

**Male genitalia** (*n* = 3) (Fig. 4E, F). Tegumen not extend distally and shorter than uncus; uncus narrow and bifurcated at distal tip, with closely aligned arms; gnathos well-developed and bifid; valva long and narrow, dorsodistal process narrow and long, ventrodistal process widened and angled at the tip; phallus with lateral process almost equal to subzonal sheath, distal half not enlarged and serrated reaching the tip of phallus; juxta horseshoe shaped.

**Female genitalia** (Fig. 5C). Papilla analis irregularly triangular in dorsal view; lamella antevaginalis trilobed shaped, lamella postvaginalis U-shaped, with upper margin straight and densely covered with fine hairs and spines; ductus bursae thick and short; bursa copulatrix oblong and membranous.

##### Distribution.

Japan (Honshu, Shikoku and Kyushu).

#### ﻿ Ochlodes pseudochraceus

Taxon classificationAnimaliaLepidopteraHesperiidae

Zhu, Fan & Wang sp. nov.

A01D53A0-41D9-57F2-9B71-1B65BA810E39

https://zoobank.org/0DDA3E56-35C3-4AEB-B99C-AF9108FAE621

Figs 3I–K , 4A, B , 5A

Ochlodes ochracea : Chu et al. 2017: 421.

##### Type material.

***Holotype***: ♂. 1.VIII.2016, Simingshan, Ningbo City, Zhejiang province, P. R, China, leg. Houshuai Wang & Shuqin Ji. SCAU_He2614. ***Paratypes***: 1♂. 1♀. SCAU_He2637 (female) and SCAU_He2676 (male) with the same data as holotype.

##### Diagnosis.

Spots in spaces R_3_–R_5_ on forewing upper side radial and far from discocellular vein. Male genitalia: lateral process of phallus with distal half gradually enlarged, with a row of small spines. Female genitalia: upper margin of lamella postvaginalis straight.

##### Description.

Forewing length 15 mm in males and 14 mm in females (Fig. 3I-K). Antenna longer than half length of forewing. Labial palpi, second segment porrect and covered with long brown hairs, third segment short.

**Male** (Fig. 3I, J). Forewing upper side: ground color dark brown with orange-yellow spots. Spots in spaces R_3_–R_5_ short and radial, far from discocellular vein; spots in space M_1_ to CuA_2_ form a broad brand, of which spot in space M_1_ very small; cell orange-yellow; stigma black-grey, thin and long. Hindwing upper side: ground color same as forewing, central part including cell and spaces Rs-CuA_2_ orange-yellow. Wing under side: ground color yellow-brown, with spots yellow, and blurred.

**Female** (Fig. 3K). Different from male in reduced spots in spaces R_3_ and M_1_, only a pair of small cell spots present on forewing; cell spot and spots in spaces Rs-CuA_2_ short and small on hindwing.

**Male genitalia** (Fig. 4A, B). Tegumen slightly shorter than uncus; uncus bifurcated at distal tip with closely aligned arms; gnathos well developed and bifid; valva long and broad, dorsodistal process triangular, ventrodistal process broad and blunt, rounded with small spines at apex, posterior angle ossified but not prominent; lateral process of phallus long, gradually enlarged in distal half, with a row of small spines along dorsal margin; juxta horseshoe shaped.

**Female genitalia** (Fig. 5A). Papilla analis irregularly triangular in dorsal view; lamella antevaginalis trilobed shaped, lamella postvaginalis U-shaped, with upper margin straight and densely covered with fine hairs and spines; ductus bursae thick and short; bursa copulatrix oblong and membranous.

##### Etymology.

The scientific name is a masculine adjective and derived from the Greek word *pseudes* (meaning false) and the species name *ochraceus*, referring to their similarity.

##### Distribution.

China (Zhejiang, Anhui).

#### ﻿ Ochlodes cryptochraceus

Taxon classificationAnimaliaLepidopteraHesperiidae

Zhu, Fan & Chiba sp. nov.

6C8D3F08-5632-5D97-9824-2486DC7E5EE2

https://zoobank.org/65EBAE39-0BDC-4829-BCA3-E80C953E3704

Figs 3L–N , 4C, D , 5B

Ochlodes ochracea : Chiba and Tsukiyama 1996: pl 1 figs 20, 22; Cai et al. 2011: 312; Yuan et al. 2015: 513; Wu and Hsu 2017: 1396.

##### Type material.

***Holotype***: ♂, SCAU_He2605, 1.VI.2019, Shennongjia, Hubei province, P. R, China. ***Paratypes***: 1♂, SCAU_He2678, 20.VI.2021, Miaowangshan, Baoji, shaanxi province, P. R, China, leg. Liping Zhou; 1♂, SCAU_He2680, 13.VI.2018; 2♂, 6.VII.2018; 1♂, 10.VI.2011, Liukan, Hanzhong, Shaanxi province, leg. Liping Zhou; 1♂, 19.VI. 2018; 1♂, 20.VI. 2018; 1♀, 6.VII.2018, Miaowangshan, Baoji, Shaanxi province, leg. Liping Zhou; 1♂, 10.VII.2011; 1♀, SCAU_He2632, 6.VI.2018; 1♀, 10.VII.2011, Heilongtan, Yulin, Shaanxi province, leg. Liping Zhou; 1♂, 12.VI.2018, Huangguan, Ankang, Shaanxi province, leg. Liping Zhou; 1♂, 24.VI.1993, Wanhuashan, Yanan, Shaanxi province, (HC); 1♂, 5.VII.1993, Qinlin, Shaanxi province, (HC); 1♀, SCAU_He2677, 1.VI.2019, Shennongjia, Hubei province; 1♂, 1.VII.2022, Mianyang, Sichuan province; 1♂, Yunnan province, P. R, China, leg. Xiaoling Fan & Min Wang.

##### Diagnosis.

Spots in spaces R_1_–R_5_ on forewing upperside long radial, reaching discocellular vein. Male genitalia: lateral process of phallus only distally enlarged. Female genitalia: upper margin of lamella postvaginalis slightly concave.

##### Description.

Forewing length 15 ± 0.5 mm in males and 14–15 mm in females (Fig. 3L–N). Antenna longer than half length of forewing, and black and white stripes extend to club.

**Male** (Fig. 3L, M). Forewing upper side: ground color dark brown with orange-red spots. Spots in spaces R_1_–R_5_ long radial and connected to discocellular vein; spots in spaces M_1_–CuA_2_ form a broad band, of which spot in space M_1_ very small. Cell orange-red; stigma black-grey and thick. Hindwing upper side: ground color same as forewing, central part including cell and spaces Rs-CuA_2_ orange-red. Wing under side: ground color red-brown, spots yellow and blurred.

**Female** (Fig. 3N). Different from male in reduced spots in spaces R_1_, _2_ ; only a pair of small cell spots present on forewing upper side; cell spot and spots in spaces Rs-CuA_2_ orange-yellow on hindwing.

**Male genitalia** (Fig. 4C, D). Tegumen slightly shorter than uncus; uncus bifurcated at end tip, with closely aligned arms; gnathos well-developed and bifid; valva long and narrow, dorsodistal process narrow and blunt, ventrodistal process irregular rectangular, and rounded with small spines at apex; lateral process of phallus long and distally enlarged with two rows of small spines; juxta horseshoe shaped.

**Female genitalia** (Fig. 5B). Papilla analis irregularly triangular in dorsal view; lamella antevaginalis trilobed, lamella postvaginalis U-shaped, with upper margin slightly concave and densely covered with fine hairs; ductus bursae thick and long, and bursa copulatrix oblong and membranous.

##### Etymology.

The scientific name is a combination of the prefix *crypt* (meaning hidden) combined with the species name *ochraceus*, which refers to the cryptic species of *ochraceus*. The name is a masculine adjective.

##### Distribution.

China (Shaanxi, Gansu, Hubei, Sichuan, Yunnan).

## Supplementary Material

XML Treatment for Ochlodes ochraceus

XML Treatment for Ochlodes rikuchina

XML Treatment for Ochlodes pseudochraceus

XML Treatment for Ochlodes cryptochraceus

## ﻿Additional information

### Author ORCIDs

Lijuan Zhu https://orcid.org/0000-0002-4525-7438

Yongxiang Hou https://orcid.org/0000-0002-4802-9406

Hideyuki Chiba https://orcid.org/0000-0001-9060-6441

Yohei Osada https://orcid.org/0000-0002-0179-9622

Sergey Yu. Sinev https://orcid.org/0000-0002-2467-5403

Min Wang https://orcid.org/0000-0001-5834-4058

Xiaoling Fan https://orcid.org/0000-0002-1176-7667

### Data availability

All of the data that support the findings of this study are available in the main text.
